# Reflections on the Regeneration of Bones; Accompanied with a Case of Necrosis of the Tibia, Comprising the Whole Circumference and Length of the Bone, with Integrity of the Medulla and of the Greater Part of the Periosteum

**Published:** 1820-08

**Authors:** E. Lebel

**Affiliations:** Paris.


FOR THE LONDON MEDICAL AND PHYSICAL JOURNAL.

### *Reflections on the Regeneration of Bones; accompanied with a Case of Necrosis of the Tibia, comprising the whole circumference and length of the Bone, with integrity of the Medulla and of the greater part of the Periosteum.* By E. Lebel, M.D. of Paris.

THE opinion generally admitted since Duhamel, and especially since the experiments of Troja, on the mode of formation of the sequestrum in necrosis of the long bones, and on the regeneration of bones by means of the periosteum, is well known; an opinion which has lately been opposed by MM. Richerand and Léveille. According to this view of the subject, which is confirmed by the observations of the greater number of practitioners, and by the experiments of modern physiologists, when the body of a long bone dies, the periosteum, if it remain healthy, is gradually detached from it, and a substance of a gelatinous appearance is effused in the interval. This substance, which is at first soft and almost liquid, acquires more consistence from day to day: blood-vessels are developed in it; it passes into a cartilaginous state, and then to that of bone; and thus constitutes a new bone, enclosing the old one in its interior, and pierced with several holes. These openings give passage to purulent matter, the formation of which ordinarily accompanies these phenomena, and which appears to be one of the means employed by nature to destroy gradually, or to expel, the sequestrum. Almost all the partizans of this theory seem to believe that the death of the medullary substance always accompanies that of the bone; for they are either silent respecting the fate of this substance, or, if they speak of it, it is to say that its death solely has produced the necrosis, and that, at the end of a greater or less length of time after the expulsion of the sequestrum, a new medullary substance is found in the new bone, that is also of new formation.

Richerand and Léveille have only adopted an opinion, already advanced by Brun, in a memoir read to the Royal Academy of Sciences of Toulouse in 1781; and victoriously refuted, in 1782, by David, a surgeon of Rouen. According to those two writers, necrosis of a long bone always depends on the death of the periosteum or of the medullary membrane, or of both. In either of the first two cases, the mortification comprises only the half, or thereabouts, of the thickness of the bone, corresponding with the membrane which has lost its vitality; in the last case, it extends throughout the whole thickness of the bone. If the osseous cylinder has continued to live in its middle, externally or internally, this part becomes soft, fleshy granulations (cellulous and vascular) are developed between it and the dead portion of bone, the latter is by degrees entirely insulated, and the limb preserves its ordinary length, without any osseous regeneration having taken place. But if the necrosis, which comprises the whole thickness and circumference of the bone, could not be followed with a similar result, and ossification of the periosteum being absolutely impossible if the limb is formed of a single bone, it is shortened in proportion to the length of the sequestrum.

Without wishing to reproduce here the judicious, and I think unanswerable, objections which the partizans of the old theory have made to this doctrine, I shall solicit attention to a case which is inexplicable by the former, and which does not appear to have been noticed by any practitioner previously to M. Boyer. It is that of a long bone being deprived of its vitality throughout its whole circumference and thickness, with simultaneous destruction of the periosteum, whilst the medullary substance continued to live.

M. Boyer, comparing the case in question to that in which the scapula is affected with necrosis throughout its whole thickness, whilst life continues to exist in the periosteum of its interior surface, pretends that an effusion of gelatinous matter will then take place on the surface of the medullary membrane, similar to that which is deposited on the inner surface of the periosteum when the latter member alone retains its vitality. But, has he not gone further here than simple analogy authorizes, in attributing to the medullary substance functions exactly the same as those we find executed by the periosteum in other instances. I do not believe that such an identity of functions has hitherto been proved by a single pathological observation; and the author does not himself relate any fact in support of a proposition that cannot be rationally admitted without positive proof.

Without wishing to deny absolutely, in man, the possibility of regeneration of bone by means of the medullary membrane, which the experiments of Troja seem to have placed beyond doubt in respect to birds, I shall remark, that, if circumstances happen in this way, the new-formed bone would be contained in the old one, which would entirely surround it. M. Boyer says that at a more or less remote period the sequestrum will become separated, being displaced by the granulations on the new one; but he does not state in what manner its expulsion can take place. Will the new bone, on developing itself, make the old one split off from it; and will the fragments of the latter be removed by suppuration? Will the process just mentioned destroy one of the sides of the sequestrum, so that it may be detached without being retained by the living bone, as it will be seen it had begun to do in the patient whose case will be hereafter related? Or, lastly, will the new bone, against all probability, itself offer a solution of continuity, to give passage to that which has ceased to live? When an author, so well known for his severe exactness, omits details of this kind, we may be permitted to suppose that he has not himself had an opportunity of observing the process of nature on such an occasion.

The following case will, it appears to me, if not entirely decide the question about the regeneration of bone by means of the medullary membrane, at least render this mean of osseous reproduction very doubtful, and lead to a close examination of it by surgical practitioners and physiologists.

MAURICE DULAC, ten years of age, the son of a gardener of Paris, assisting, as far as his strength would permit, his father in his labours, experienced, towards the end of March 1818, severe pain in the left knee, which soon increased in size. On being called to him, two or three days after the appearance of these symptoms, I ordered ten leeches to be applied about the diseased joint, which was afterwards covered by an emollient and anodyne poultice. A few days afterwards, the swelling was diminished, and a slight fluctuation, which had been felt above the patella, had disappeared, when suddenly the leg became red, tense, and painful. A new and very extensive fluctuation was soon perceived at the anterior and middle part of the leg; and an incision, of two inches in extent, gave issue to some pus of a good appearance: it was at the same time easy to perceive that the bone, which was bare at the bottom of the wound, had lost its vitality. I told the parents that a bony lamina would be detached from it at the end of a few months; and from this time I saw the patient only after intervals of twelve or fifteen days. However, there was a serous suppuration in moderate abundance; the detachment of the skin extended to a considerable distance above and below the incision, and the leg continued much enlarged, not only from the swelling of the soft parts, but also from an increase in the size of the bones, the ends of which, and especially those of the fibula, formed a projection under the skin much greater than that from those of the right leg. On the 11th of July I felt the bone, at the bottom of the wound, move easily under the fingers; and on the following day I prolonged the incision, from above downwards, as far as the extremities of the part affected with necrosis. From this time, the dead piece of bone, being daily moved, permitted of more free and extensive motions from day to day; and it gave way at length, on the 22d, to my efforts to withdraw it. I seized it by the inferior extremity, passing one of the blades of a strong pair of forceps into the medullary canal; and, after several rotatory movements, I raised my hand, making the end of the bone which I held describe the arc of a circle, the centre of which was constituted by the other extremity of the bone. The medullary substance was probably torn towards its lower part by the latter motion; for, as I proceeded in the extraction, I perceived that the sequestrum comprised the whole of the circumference of the bone, and I observed the medullary substance pass out of the cylinder which I brought away, continuous above with the part contained in the superior extremity of the tibia. After having sustained it for some seconds on my hand, and having recognized that its surface, of a flesh colour, presented nothing which indicated the commencement of ossification, I placed it gently in the place which it should occupy; and the wound, covered with blood, was washed and dressed in a simple manner. The piece of bone which I extracted was near five inches in length, (about five inches and a half English measure,) and comprised in one portion of its extent the whole thickness and circumference of the tibia. Not knowing to what point nature had proceeded for the replacement of this bone, the limb was placed in the apparatus for fracture, where it remained for more than a month, although I had ascertained, at the first dressing, that the middle and anterior part of the leg presented already a considerable degree of solidity, owing, without doubt, to ossification of the periosteum. On the ensuing days the wound gradually contracted, continuing to furnish pus in moderate abundance, and which imparted a strongly-marked green colour to the bandages. The boy, after having limped for a long time, although the diseased limb was exactly of the same length as the other, walks now without sensible lameness, and joins in all the labours and sports of his brothers without ever complaining of any pain; but the anterior and middle part of the leg still presents a considerable depression, which is the more strongly marked because the upper and lower ends of the tibia are more voluminous than in the ordinary state. It is now seventeen months since the sequestrum was extracted, and yet the cicatrix is not completely formed. The wound is covered with thin scales, from under which there oozes some purulent matter, commonly hardly sensible, but it is increased when the boy engages in any inordinate labour. Bony resistance is clearly felt on each side; but the middle of the leg seems still to be a little soft to the touch, although moderate pressure does not cause any pain. With regard to the sequestrum, which I have now before me, its interior and exterior surfaces are perfectly smooth, and similar in every respect to those of a healthy bone taken from a subject of the same age. The posterior surface alone presents some roughness, which appears, evidently, to be the result of the process which nature had commenced to prepare for its expulsion. The half of that surface is indeed already destroyed throughout its whole thickness; and, if the dead bone had remained longer in contact with the neighbouring parts, I do not doubt but that it would have been entirely corroded, and that, on this side of the cylinder being opened, it would have gradually come away, without doing injury to the medullary substance. The last remark, in connection with the observations of M. Dupuytren, proves that, in a similar case, we should not be in haste to make the extraction of the sequestrum.

If I do not deceive myself, the case above related, which is one that may be expected to occur frequently in large hospitals, proves,

1. That necrosis of the whole circumference and thickness of a long bone may happen without destruction of the periosteum and of the medullary membrane, although Richerand and Léveille pretend, after Brun, that the death of those membranes is indispensable, in order that it may take place.

2. That if, after this necrosis of the whole thickness of the bone, the medullary membrane, remaining unaffected, secretes (as M. Boyer states) a gelatinous matter which is to be at length interspersed with calcareous phosphate, this new ossification is here effected much more slowly than on the surface of the periosteum. Nearly three months had elapsed from the time when the bone had first ceased to live and that at which I extracted it, and nothing yet indicated a commencement of ossification at the surface of the medullary membrane, which appeared to enjoy vigorous life. So long a time, during which nature had not yet done any thing on the part of the medullary membrane towards the formation of a new bone, whilst osseous formation of considerable firmness already existed at the surface of the periosteum, appears to me to authorize a strong presumption against the opinion which attributes, in this respect, the same functions to the medullary membrane as those possessed by the periosteum. M. Cruveilheir states, in his

Essay on Pathological Anatomy, the repugnance he has to attribute identical functions to tissues so different in their organization; but this last circumstance would be a very weak objection to the opinion of M. Boyer, since M. Cruveilheir himself has seen even muscles, very different without doubt from the periosteum, become ossified in birds, in order to supply the bone which he had destroyed in his experiments on them. Direct observation, then, can alone reply to this question; and I believe but little is left to be desired in this respect, after the case I have just related.

I may be permitted to add to an incontestible fact, a consideration which appears to me to be of some importance. It is undoubtedly advantageous for a limb to preserve its natural length; but how much more important is it, that it should continue to enjoy its power of motion. Now we may well conceive how the attachments of the tendons, the aponcuroses, and ligaments, are preserved, when it is the periosteum which serves as the basis for the new bone; but we cannot comprehend equally well how these fibrous parts, many of which terminate in muscles which tend to draw them from the place of their insertions, after having been detached from the exterior bone affected with necrosis, should come to be fixed to exactly the corresponding parts of the internal bone of new formation. This would not then have with the moving powers the connections necessary for the exercise of their functions; and nature, (let me be permitted to use this expression,) after replacing with much trouble the sequestrum, and thus preserving the length of the limb, would have failed in the most important object, and hardly any benefit would have been derived from her efforts.

This consideration adds, I confess, some force, in my mind, to the opinion which denies the faculty of producing a new bone to the medullary membrane as well as to the periosteum; and, on reflecting on the results of the twelfth experiment of Troja, (*De novorum Ossium generatio experimenta*, Parisiis, 1775,) which, on a first consideration, seems exactly contrary to this view, I have thought that this opposition might be more apparent than real. Perhaps it would be sufficient to make it disappear, to sacrifice the animal at a more distant period from the time of the destruction of the periosteum, than was done by the Neapolitan physiologist. I shall be assiduous, whenever circumstances will permit, to interrogate nature on this important point; and, whether the result of my researches be favourable or adverse to the notions I here advance, if it appears to me to be worthy of engaging the attention of physicians, I shall not hesitate in its publication.

